# Retraction Note: Application of 4% chlorhexidine to the umbilical cord stump of newborn infants in lower income countries: a systematic review and meta-analysis

**DOI:** 10.1186/s40748-020-00118-y

**Published:** 2020-06-16

**Authors:** Aklilu Abrham Roba, Maleda Tefera, Teshager Worku, Tamirat Tesfaye Dasa, Abiy Seifu Estifanos, Nega Assefa

**Affiliations:** 1 College of Health and Medical Sciences, Haramaya University, Harar, Ethiopia; 2 School Of Public Health, Addis Ababa University, Addis Ababa, Ethiopia

**Retraction Note: Matern Health Neonatol Perinatol 5, 16 (2019)**

**https://doi.org/10.1186/s40748-019-0111-y**

This article [1] has been retracted by the Editors in Chief.

After publication, errors were identified and brought to the attention of the authors. These included errors in data abstraction from the included trials and the erroneous pooling of data, using meta-analysis, which had been derived via different analytic methods within the primary trials (e.g. intention-to-treat trial results were used from some of the included trials and per-protocol results from others).

These errors have rendered the conclusion of primary outcome (a reduction in neonatal mortality in lower income countries following topical application of 4% chlorhexidine to the umbilical cord stump) and its recommendation invalid while secondary outcomes [omphalitis and cord separation time] are not affected.

They also affected Figs. 2 and 3. The authors have been given an opportunity to re-submit their work once the errors are corrected.

Authors Aklilu Abrham Roba, Tamirat Tesfaye Dasa, Nega Assefa, Teshager Worku and Abiy Seifu Estifanos agree with this retraction. Author Maleda Tefera did not respond to the correspondence from the journal regarding this retraction notice.
